# Targeted breast cancer therapy by harnessing the inherent blood group antigen immune system

**DOI:** 10.18632/oncotarget.14746

**Published:** 2017-01-19

**Authors:** Wei Han, Wei Li, Xiaoying Zhang, Zhonghua Du, Xiaoliang Liu, Xin Zhao, Xue Wen, Guanjun Wang, Ji-Fan Hu, Jiuwei Cui

**Affiliations:** ^1^ Stem Cell and Cancer Center, First Affiliated Hospital, Jilin University, Changchun, Jilin 130021, China; ^2^ Stanford University Medical School, Palo Alto Veterans Institute for Research, Palo Alto, CA 94304, USA

**Keywords:** cancer therapy, gene therapy, blood group antigen, immune response, immunogenic cell death

## Abstract

Cancer gene therapy has attracted increasing attention for its advantages over conventional therapy in specific killing of tumor cells. Here, we attempt to prove a novel therapeutic approach that targets tumors by harnessing the blood antigen immune response system, which is inherently present in patients with breast cancers. Breast cancer MDA-MB-231 cells expressed blood group H antigen precursor. After ectopic expression of blood group A glycosyltransferase, we found that the H precursor was converted into the group A antigen, appearing on the surface of tumor cells. Incubation with group B plasma from breast cancer patients activated the antigen-antibody-complement cascade and triggered tumor cell killing. Interestingly, expression of blood A antigen also reduced tumorigenesis in breast cancer cells by inhibiting cell proliferation, migration, and tumor sphere formation. Cell cycle analysis revealed that cancer cells were paused at S phase due to the activation of cell cycle regulatory genes. Furthermore, pro-apoptotic genes were unregulated by the A antigen, including BAX, P21, and P53, while the anti-apoptotic BCL2 was down regulated. Importantly, we showed that extracellular HMGB1 and ATP, two critical components of the immunogenic cell death pathway, were significantly increased in the blood A antigen-expressing tumor cells. Collectively, these data suggest that blood antigen therapy induces specific cancer cell killing by activating the apoptosis and immunogenic cell death pathways. Further translational studies are thereby warranted to apply this approach in cancer immuno-gene therapy.

## INTRODUCTION

Despite the development of numerous new therapeutic strategies, many cancers remain incurable in most patients, particularly when the diseases are diagnosed at advanced stages. Conventional chemotherapeutics, though often effective, are highly toxic due to the lack of selectivity to cancer cells [1]. Recent efforts have thus focused on developing therapies that target genes that are specifically expressed in tumors [2–5]. Among these approaches, tumor suicide gene therapy has attracted increasing attention for its potential advantages over conventional therapy [6–8]. Tumor-specific killing can be induced by activation of therapeutic genes in targeted tumor cells under the control of a tumor-promoter. Such transfected tumor cells may be capable of inducing a “bystander effect”, triggering the death of neighboring untransfected cells [9]. One approach is to utilize gene-directed enzyme prodrug therapy. HSVTK/GCV and CD/5FCB are the best characterized systems [10, 11]. Despite its advantages, however, suicide gene therapy exhibits limited efficiency due to poor targeting potential, suicide gene expression, catalytic activity of the enzyme product, and killing effects [12]. Owing to the limited antitumor activity, drugs using this therapeutic approach have rarely advanced to market.

As demonstrated recently, success in tumor gene therapy has been achieved by harnessing endogenous immune mechanisms [13, 14]. T cells can be armed to express a chimeric antigen receptor (CAR) that recognizes a specific antigen molecule on tumor cells [15–18]. This approach has demonstrated potent clinical efficacy as it can induce remissions in patients with advanced leukemia [19–21]. The human blood group antigen-antibody system is another powerful inherent immune mechanism. Human blood is grouped according to the ABO blood typing system, depending on the expression of antigens on red blood cells and antibodies in the plasma. Immune-mediated hemolytic transfusion reactions occur when incompatible blood products are transfused into a patient's circulation, triggering a cascade response from the patient's immune system [22, 23].

We wished to learn whether this inherent immune system could be exploited to target tumors in patients by replacing tumor suicide genes in conventional suicide gene therapy system with an incompatible blood group enzyme. Tumor-specific expression of a blood group enzyme would convert the endogenous H precursor into an incompatible blood antigen on the surface of tumor cells. Antibodies in the circulation of cancer patients would specifically recognize and lyse tumor cells that express the incompatible blood antigen.

In this study, we explore whether ectopic expression of an incompatible blood group antigen is a practical gene therapy approach to target triple negative breast cancer MDA-MB-231 cells (abbreviated as MDA231 hereafter). Addition of plasma that contains an incompatible blood type antibody is able to specifically lyse breast cancer cells. The mechanisms underlying tumor cell killing, particularly immunogenic cell death, are also explored.

## RESULTS

### Development of the blood group antigen tumor therapy

**Figure 1:**
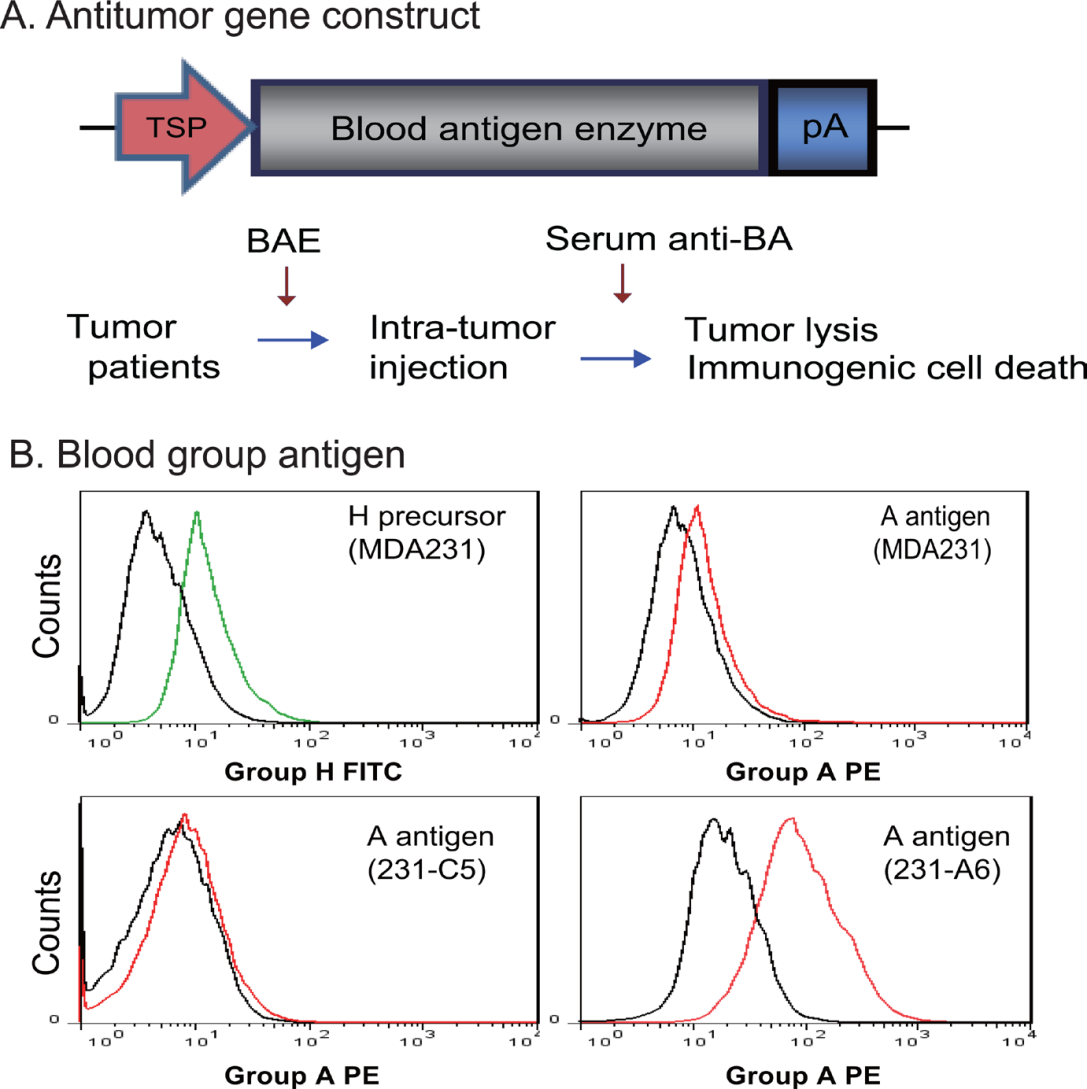
Antitumor therapy by blood group A antigen in MDA231 breast cancer cells (**A**) Schematic diagram of blood group A antigen-induced antitumor therapy. TSP: tumor-specific promoter; pA: TK poly A signal; BAE: blood antigen enzyme; Anti-BA: anti-blood antigen antibodies present in the circulation of patients with breast cancer. In tumor gene therapy, the BAE is expressed in tumors under the control of a tumor-specific promoter, leading to the presentation of the “incompatible” blood antigen on the surface of tumor cells. This will trigger the immune response by the endogenous anti-blood antigen antibodies in the circulation of the cancer patients and kill tumor cells through the mechanism of immunogenic cells death. (**B**) Conversion of the endogenous H antigen into group A antigen in MDA231 breast cancer cells. 231-C5: MDA231 control cells that were stably transfected with the empty lentiviral vector; 231-A6: MDA231 cells that were transfected with blood group A transferase. The black line in the histogram represents the negative isotype control; the green line represents the fluorescence intensity of the anti-group H FITC; and the red line represents the anti-group A PE. Note the abundant A antigen expression in 231-A6 cells.

Conventional tumor suicide gene therapy induces tumor killing by specific activation of therapeutic genes in targeted tumor cells under the control of a tumor-specific promoter. However, this gene therapy approach has a limited antitumor activity. We proposed to test a novel tumor gene therapy approach by harnessing the potent blood group antigen-antibody immune cascade system (Figure 1A). In this system, the suicide gene is replaced by a blood antigen enzyme (BAE). Tumor-specific expression of the BAE will convert the endogenous H antigen precursor into a specific blood group antigen. The appearance of the incompatible blood group antigen on the surface of tumor cells is then recognized by the anti-blood group antibodies that are abundantly present in the circulation. This will trigger the immune cascade reaction and induce tumor-specific cell lysis, providing a molecular basis for targeted tumor gene therapy.

In this proof-of-concept study, we constructed a lentiviral vector containing the ABO gene that encodes the A glycosyltransferase enzyme. When expressed in breast cancer MDA231 cells, the enzyme adds either N-acetyl galactosamine or galactose to the H antigen, converting it into the A antigen. Using flow cytometry, we found that the H antigen was ubiquitously expressed in four breast cancer cell lines (Supplementary Figure 1). MDA231 breast cancer cells expressed the H antigen precursor, but the A antigen was barely detectable (Figure 1B).

To generate s antigen expression in MDA231 tumor cells, we transfected the cells with lentiviruses carrying the group A glycosyltransferase cDNA. After puromycin selection, the 231-A6 clone cells stably expressed group A glycosyltransferase and were used for further study. FACS analysis shows that > 93% of 231-A6 cells were positive for the vector tracking marker copGFP (Supplementary Figure 2). FACS analysis showed the successful conversion of the H precursor into blood group A antigen in 231-A6 tumor cells (Figure 1B, right bottom panel). MDA231 control cell clone (231-C5), which carried the empty vector and did not express the group A antigen (bottom left panel), was used as the negative control in the study.

### Group B plasma induces cell death in the group A antigen-expressing MDA231 cells

To examine the potential of the blood antigen gene therapy approach, we collected plasma samples from breast cancer patients with type B blood. We hypothesized that the group B plasma, which contains the endogenous anti-A antibody, would be able to trigger immune system and specifically kill 231-A6 tumor cells that carry the group A antigen.

**Figure 2:**
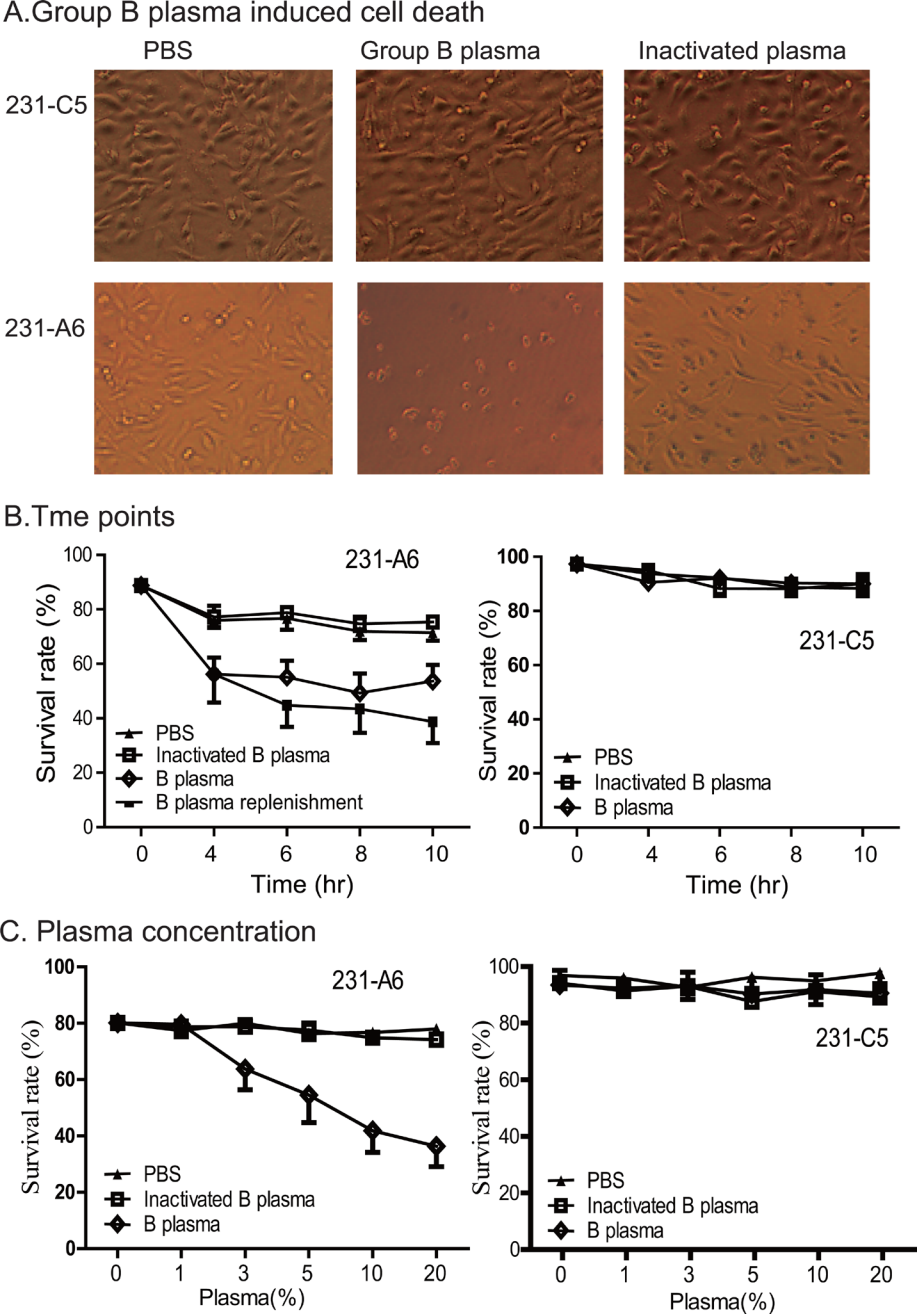
Group B plasma induces cell death in 231-A6 cells (**A**) Group B plasma inhibited the growth of 231-A6 breast cancer cells. The A antigen-expressing 231-A6 cells were treated for 24 hours with PBS, 5% human group B plasma, and 5% inactivated group B plasma, respectively. Cells carrying the empty vector (231-C5) were used as the control. (20X objective, Olympus, Japan). (**B**) Quantitation of cell death rate at different time points. 231-A6 tumor cells were treated with 5% B plasma. In the replenishment group, fresh 5% B plasma was added into the cells at 4, 6, and 8 hours. (**C**) Effect of group B plasma concentration on cell death in 231-A6 cells. Cell survival was quantitated by FACS at 24 hours following B plasma treatment. 231-C5 cells were used as the control.

To define the role of this unique gene therapy system, the A antigen-expressing 231-A6 cells were divided into three groups and were treated with PBS control, 5% human group B plasma, and 5% heat-inactivated group B plasma, respectively. By inactivating group B plasma, we hoped to define if other plasma components, such as complement, are involved in the anti-tumor effect. As compared with the PBS control, treatment with group B plasma induced cell shrinkage and reduction in cell number in 231-A6 cells (Figure 2A, left vs middle panels). However, these changes were not seen in cells treated with inactivated plasma (right panel), suggesting the requirement of active components in group B plasma.

We then used flow cytometry assays to examine cell death at different time points of treatment. There was a significant early increase in cell death in the B plasma-treated group as compared with those in the inactivated plasma group and the PBS control group. However, no further increase in cell death was observed as the treatment duration was prolonged after 4 hours, suggesting the depletion of active components in the B plasma. To support this hypothesis, we added fresh B plasma at 4, 6, and 8 hours. As expected, more cells died after replenishment of B plasma (Figure 2B, left panels). No significant changes in cell death were seen in 231-C5 cells that carried the empty vector (Figure 2B, right panel).

Next, we varied the concentration of the plasma, from 1% to 20%. Flow cytometry assay was used to examine cell death following the B plasma treatment. There was a dose-dependent effect of B plasma treatment after 24 hours following plasma treatment (Figure 2C, left panel). This cell-killing was not observed in the heat-inactivated B plasma group, suggesting that heating inactivates plasma components, like complement, that are required for tumor killing. These changes were not observed in 231-C5 control cells that carried the empty vector (Figure 2C, right panel).

To confirm the specificity of type B plasma, we depleted the anti-A antibody in the group B plasma. After depletion of the anti-group A antibody, type B plasma no longer induced cell death in 231-A6 cells (Supplementary Figure 2A). Similarly, replacement of group B plasma with group A plasma did not affect cell survival (Supplementary Figure 2B). These data indicate a critical role of the A antibody in group B plasma in triggering tumor cell death.

### Treatment with group B plasma reduces cell proliferation and migration in 231-A6 cells

**Figure 3:**
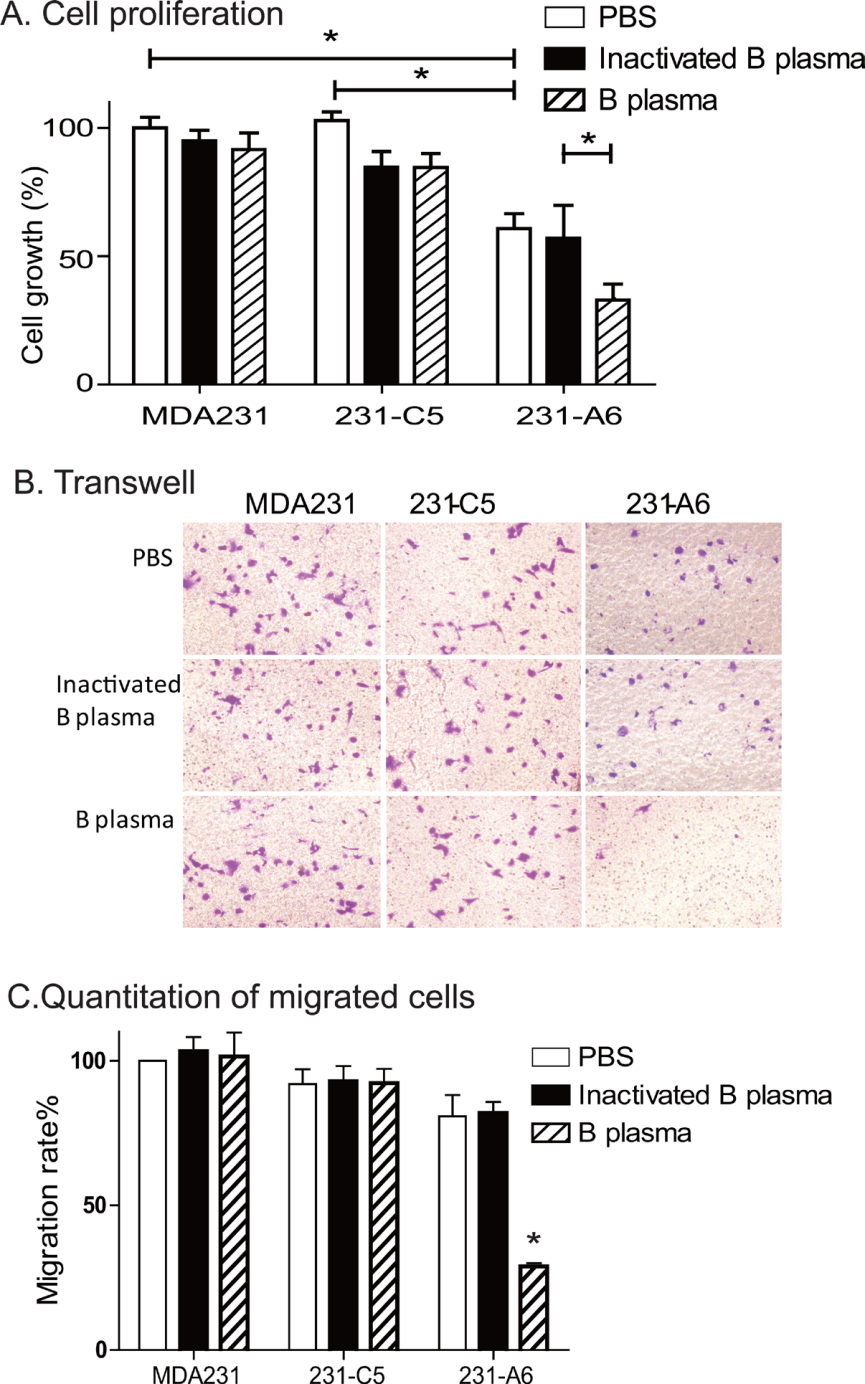
Group B plasma reduces cell proliferation and migration (**A**) Cell proliferation as measured by WST-1 assay. Cells were treated with 5% B plasma for four hours. Forty-eight hours following plasma treatment, cells were collected for the measurement of cell growth. Inactivated group B plasma was used as the assay control. *p < 0.05 between the inactivated B plasma and the B plasma groups. (**B**) Cell migration as measured by the transwell assay. Cells (5 × 10^3^ cells/well) were incubated with B plasma for 4 hours and were tested for migration in a transwell plate. Migrated cells were stained with crystal violet (20× objective). (**C**) Quantitation of the migrated cells. Migrated cells were counted in five random fields and averaged for analysis. *p < 0.05 between the inactivated B plasma and B plasma groups.

After treatment with 5% group B plasma for 4 hours, we used the WST-1 assay to quantitate cell proliferation. In the PBS control group, the survival rate of B plasma group and inactivated group was 94.9% and 91.6%, respectively. For 231-C5 vector control cells, the survival rate of B plasma group and inactivated group was 84.7% and 84.5%. There was no significant difference between the B plasma group and the inactivation group in MDA231 and 231-C5 cells. However, for 231-A6 tumor cells that express the blood group A antigen, the survival rate in the B plasma group was significantly lower than that in the inactivated B plasma group (32.9% vs 60.8%; *p* < 0.05) (Figure 3A).

A transwell assay was then used to examine the effect of group B plasma treatment on cell migration (Figure 3B, 3C). In 231-C5 tumor cells that carry the empty lentiviral vector, there were no statistical differences in migrated cell number, with 29.0, 29.4 and 29.2 in PBS control, inactivated B plasma and group B plasma groups, respectively. In 231-A6 cells that express the group A antigen, however, there was a reduction in cell migration in the plasma group (*P* < 0.01). It should be pointed out that as B plasma also reduced cell survival in 231-A6 cells, it is hard to distinguish if the reduction is derived from the decreased cell mobility, or the reduced cell number, or both.

### Group B plasma induces apoptosis in 231-A6 tumor cells

**Figure 4:**
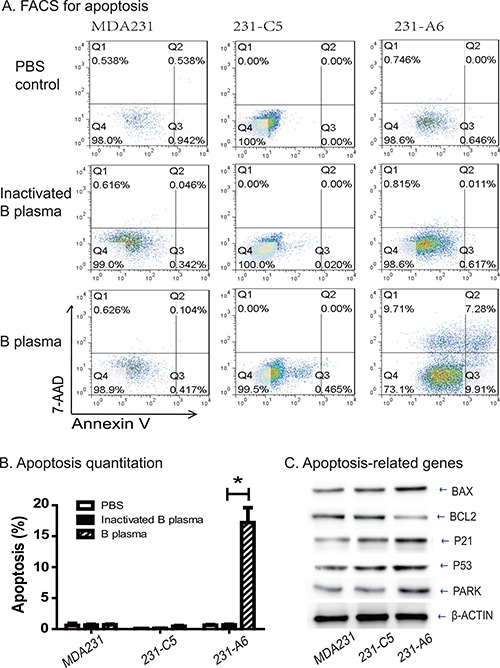
Group B plasma induces cell apoptosis in 231-A6 cells (**A**) Apoptosis as measured by FITC Annexin V-FACS assay. (**B**) Quantitation of cell apoptosis. *p < 0.05 between the inactivated B plasma and B plasma groups. (**C**) Western blot analysis of cell cycle-related proteins. β-Actin was used as the control.

To delineate the mechanism underlying the B plasma therapy, we examined apoptosis after treatment of tumor cells with 5% B plasma. For MDA231 control cells, the apoptosis rates in the PBS group, inactivation B plasma group and B plasma group were 0.59%, 0.67% and 0.69%, respectively. For 231-C5 control cells, the apoptosis rates were 0.10%, 0.12% and 0.47% in three groups. For 231-A6 cells, however, the apoptosis rates were 0.62%, 0.67% and 17.19% in the three groups (Figure 4A, 4B). These data suggest that treatment of 5% plasma B for 4 hours induces statistically significant higher apoptosis in 231-A6 cells than those in the inactivated plasma group and the PBS control group (*P* < 0.05). In addition, we also observed cell necrosis in treated cells (Figure 4A, Annexin V-negative/7ADD-positive).

We further examined the genes that are involved in the apoptotic pathway (Figure 4C). Expression of the group A antigen activated several of these genes, including BAX, P21, P53, and PARK. In contrast, the anti-apoptotic BCL2 was reduced in 231-A6 cells. Thus, B plasma therapy activates the apoptotic pathway in MDA231 tumor cells.

### Group A antigen reduces the tumor potential in MDA231 cells

**Figure 5:**
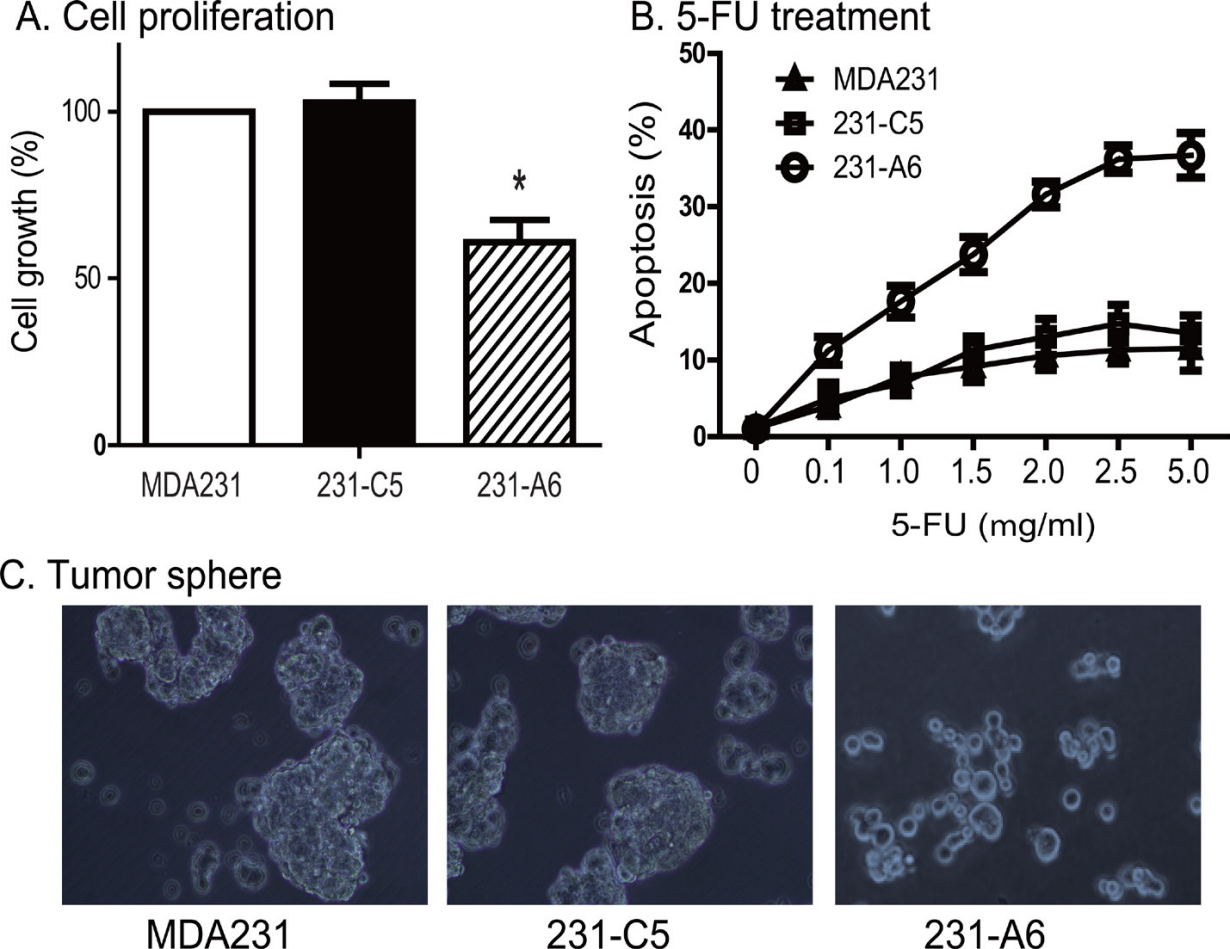
Reduced tumor potential of 231-A6 cells after blood group antigen A transfection (**A**) Blood group A antigen inhibits cell proliferation in MDA231 cells. Cell viability was quantitated by WST-1 assay. *p < 0.05 compared with the MDA231 control and 231-C5 vector control groups. (**B**) Group A antigen potentiates the therapeutic effect of 5-FU in MDA231 cells. Cells carrying the empty vector control and group A antigen were treated with different doses of 5-FU. The data are expressed as the mean ± standard deviation of three independent experiments, which quanitfy apoptosis rate by flow cytometry. **p < 0.01 compared with the control groups. (**C**) Reduced formation of tumor spheres in the A antigen-expressing MDA231 cells, using 20× objective (Olympas, Japan). Expression of the group A antigen blocks the formation of tumor sphere.

It is interesting to note that expression of blood group A antigen, even in the absence of group B plasma, also inhibited cell growth. The average survival rate was reduced to 60.8% in 231-A6 cells as compared with MDA231 (100%) and 231-C5 tumor cells (108%) (*P* < 0.01, Figure 5A). These data suggest that expression of the blood group A antigen may inhibit cell proliferation in MDA231 tumor cells.

Given the fact that group A antigen inhibits the growth of 231-A6 cells, we examined the response of MDA231 tumor cells to chemotherapy. 5-FU is an S phase specific chemotherapeutic drug commonly used in breast cancer. 231-A6 tumor cells were treated with low doses of 5-FU (0.1 to 5.0 μg/ml). We found that the group A antigen/5-FU therapy induced dose-dependent cell apoptosis (Figure 5B).

We also examined if the A antigen was able to affect the formation of tumor spheres (Figure 5C). MDA231 cells were cultured in stem cell culture medium for 1 week. As expected, both the MDA231 control cells (PBS, left panel) and 231-C5 cells (empty vector, middle panel) formed clone spheres with compact structure. However, the group A antigen-expressing 231-A6 cells failed to form spheres in the same medium (right panel).

### Group A antigen blocks MDA231 cells at S phase of cell cycle

**Figure 6:**
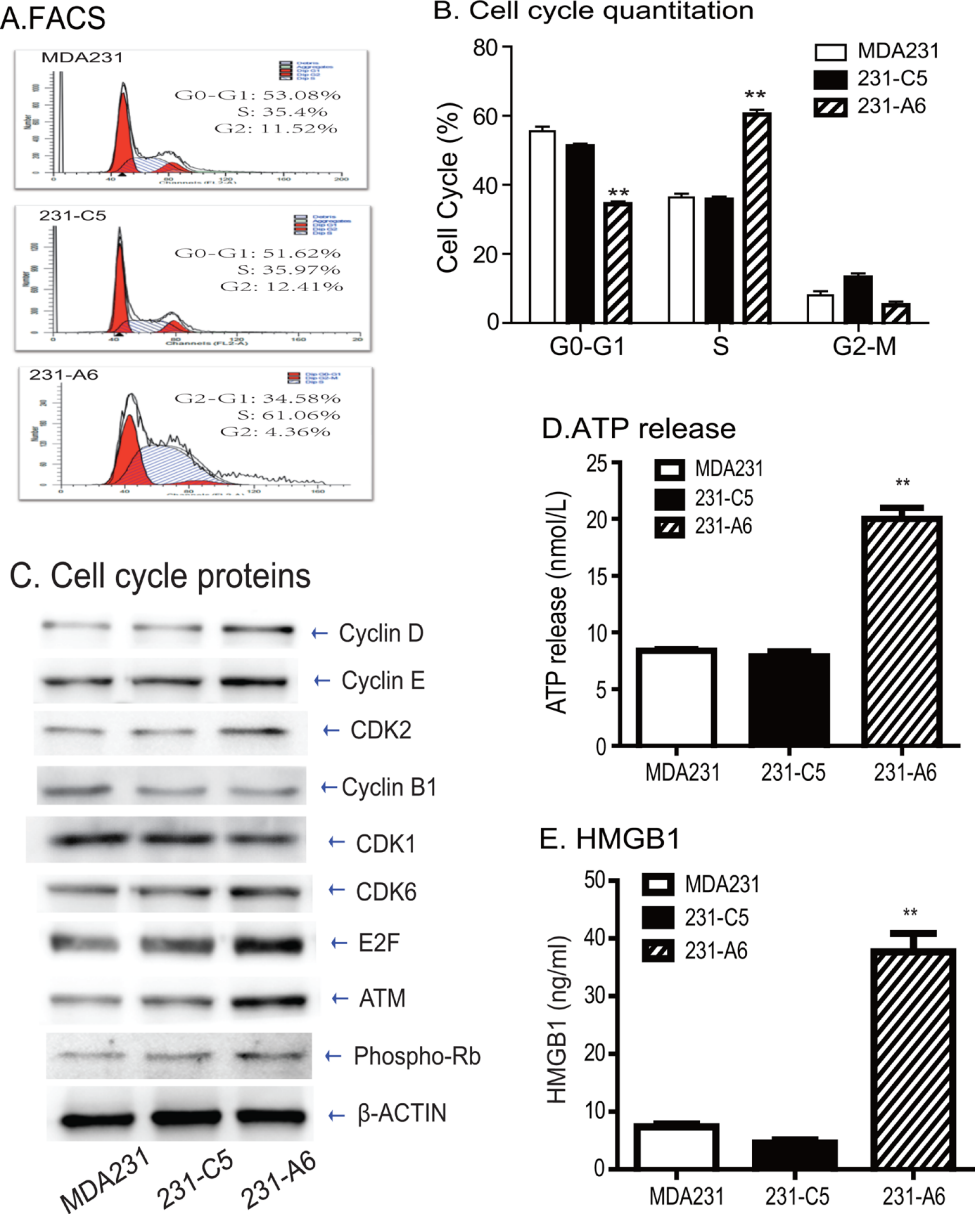
Blood group antigen induces S phase arrest and immunogenic cell death (**A**) FACS analysis of cell cycle in blood group A antigen-expressing MDA231 cells. (**B**) Quantitation of cell cycle. Note the cell pause at S phase in blood group A antigen-expressing MDA231 cells. (**C**) Western blot analysis of cell cycle-associated proteins. (**D**) The release of cellular ATP in the medium. The data are the mean ± standard deviation of three independent experiments. **p < 0.01 compared with the control groups. (**E**) Quantitation of extracellular HMGB1. **p < 0.01 compared with the control groups.

To delineate the role of blood A antigen in the inhibition of cell growth, we examined cell cycle in the A antigen-expressing MDA231 tumor cells. Using FACS, we found that there was a significant increase in S phase of 231-A6 cells compared to those in other control groups (Figure 6A). The percentage in S phase of MDA231, 231-C5 and 231-A6 cells was 35.4%, 35.9%, and 61.1%, respectively (Figure 6B, *p* < 0.01), suggesting that ectopic expression of the blood group A antigen caused MDA231 cells arrest at S phase of cell cycle. Using Western blot, we found that cell-cycle associated factors were upregulated in the group A antigen-expressing 231-A6 cancer cells, including Cyclin D, Cyclin E, CDK2, CDK6, E2F, and ATM (Figure 6C).

To further assess potential mechanisms underlying this antigen therapy, we examined whether antitumor immune activities might be involved in the therapeutic effect of the group A antigen. For this, we examined the extracellularly secreted/released ATP, a critical component in the immunogenic cell death pathway. Ectopic expression of group A antigen significantly increased the release of ATP in 231-A6 cells as compared with vector control (231-C5) and control MDA231 cells (Figure 6D). The increased release of ATP was also observed in a second breast cancer cell line MCF-7 that carried the group A antigen (Supplementary Figure 4).

HMGB1 is an abundant chromatin binding protein that exits dead cells during immunogenic cell death. We found that there was a significantly greater release of HMGB1 in 231-A6 cells than those in control MDA231 cells and 231-C5 vector control cells (*P* < 0.01, Figure 6E). Collectively, these data suggest that group A antigen may function, at least partially, by activating the immunogenic cell death pathway.

## DISCUSSION

Suicide gene therapy is an attractive development in tumor therapies. Although substantial progress has been achieved in past decades, the antitumor efficiency of gene therapy is still very limited. In this communication, we have provided proof-of-concept for a novel gene therapy approach that targets tumors by harnessing the inherent immune hemolytic reaction. Human ABO blood types are grouped by the antigens on red blood cells. “Compatible” antibodies in the plasma allow the body to recognize blood as its own. Transfusion of incompatible blood products into a patient's circulation will trigger a cascade response from the patient's immune system. We replaced the tumor suicide gene in conventional suicide gene therapy system with an enzyme that would synthesize an “incompatible” blood group. Tumor-specific expression of the enzyme would convert the H antigen precursor into an incompatible blood antigen that is present on the surface of tumor cells. Blood type antibodies that are readily present in the circulation of cancer patients will specifically lyse tumor cells that express the incomparable blood antigen.

In our proof-of-concept study, we have stably expressed a blood group A-forming enzyme in breast cancer MDA231 cells. This leads to the appearance of blood group A antigen on the surface of tumor cells. FACS analysis demonstrate the successful conversion to the group A antigen from the endogenous H antigen precursor. As hypothesized, we demonstrate that the addition of group B plasma induces an “incompatible antigen blood transfusion” cascade reaction. Incubation with the “incompatible” type B plasma triggers the immune cascade response. Consequently, the immune response induces cell apoptosis and inhibition of cell proliferation and migration in MDA231 tumor cells. Thus, this study provides the first proof of the concept that the endogenous blood group antigen-antibody immune system may be explored as a potent approach to target tumors.

The specific mechanisms underlying this blood antigen gene therapy remain to be delineated. Our initial data suggest the control of cell cycle by the B plasma treatment. It is known that cell cycle is controlled at two specific checkpoints: G1-S and G2-M phases, which are regulated by cyclins and cyclin-dependent kinases (CDKs) [24]. Transition from G1 to S phase requires the activation of cyclin D/CDK4 and cyclin E/CDK2 complexes [25, 26]. In this study, we found that the transferase A-expressing cells accumulated in S phase and arrested prior to the G2/M checkpoint. Using Western blot, we detected the altered expression of proteins involved in cell cycle regulation. Cyclin D and CDK6, two central mediators in the transition from G1 to S phase, were upregulated in the A antigen-expressing cells. On the other hand, CDK1, which is necessary for cells to enter mitosis, was down-regulated. Consequently, the entry of cells into the M phase was blocked, resulting in accumulation of cells in the S phase.

There is a cross-talk between the pathways regulating cyclins and apoptosis. In the cells that express the blood A antigen, BAX, P53, and PARK were upregulated. The upregulated P53 increases its downstream target P21, which in turn prevents the activation of the cyclin B/CDK1 complex and blocks the progression of cells through the cell cycle [27, 28]. In this study, we found that expression of the blood A antigen induced a reduction of cyclinB1 and an increase in P53. Additionally, the ATM-chk2-Cdc25 pathway is involved in G2 arrest. We also demonstrate the activation of the ATM pathway in the A antigen-expressing tumor cells. ATM further activates the P53-dependent apoptosis, accompanied by increased BAX and decreased BCL-2.

Immunogenic cell death (ICD) is a new concept that has just emerged in the past few years to delineate the pathway involved in tumor cell death related to therapeutic treatment. ICD is characterized by alterations in the composition of cell surface as well as the release of a series of soluble immune-stimulatory damage-associated molecular patterns (DAMPs) in a programmed sequence [29]. The immunogenic characteristics of ICD are mediated by DAMPs, including the secretion of ATP during the blebbing phase of apoptosis, the cell death–associated release of the non-histone chromatin protein high-mobility group box 1 (HMGB1), and the pre-apoptotic exposure of calreticulin (CRT) and other endoplasmic reticulum (ER) proteins on the cell surface [30]. Most DAMPs can be recognized by pattern recognition receptors (PRRs) [31]. Several chemotherapy compounds, like anthracyclines and oxaliplatin, are able to activate the ICD pathway in initiating antitumor immune response [32]. Oncolytic viruses also induce ICD in addition to their direct tumor cell lysis [33–36]. Likewise, radiation therapy also induces cardinal signs of ICD, including cell death and release of ATP and HMGB1 in multiple carcinoma types, including MDA231 breast cancer cells [37]. In this study, we found that blood A antigen-expressing cells exhibited a significant reduction in cell proliferation, migration, and tumor sphere formation, and exhibited increased sensitivity to the low dose of 5-FU. This tumor cell killing was in parallel with the prominent increase in extracellular ATP release and HMGB1, underscoring the critical involvement of ICD as a potential mechanism in this gene therapy approach.

It is interesting to note that expression of blood group A antigen, even in the absence of group B plasma, also reduces tumor potential, including cell proliferation, cell migration and tumor sphere formation. It is not clear what mechanisms underlie this tumor killing by blood group A antigen alone. Using FACS, we found that the blood group A antigen-expressing MDA231 tumor cells paused at S phase, in parallel with the upregulation of proteins that are associated with the control of cell cycle. As a result of S phase arrest, ectopic expression of group A antigen significantly enhances cell killing induced by very low doses of the chemotherapeutic agent 5-FU. Most importantly, the group A antigen can trigger immunogenic cell death as quantitated by the elevated release of ATP and HMGB1. Thus, we believe that this “bystander effect” is an added benefit of our glycosyltransferase gene therapy approach. It may offer a novel “one-two punch” tumor therapy for breast cancers, not only by inducing the Anti-A antigen effect but also by triggering the immunogenic cell death.

In our blood antigen therapy approach, tumor cells are transfected by a blood antigen enzyme (BAE) that converts the endogenous H antigen precursor into an “incompatible” blood antigen. It is possible that the anti-tumor effect is related either to the ectopic expression of the A antigen or to the presence of the enzyme itself. However, our data have clearly demonstrated the critical role of group A antigen in this gene approach. First, we show that depletion of the A antibody in B plasma abolishes the antitumor activity. Second, replacement of B plasma with A plasma did not inhibit cell growth in 231-A6 cells that carry the same blood antigen enzyme (Supplementary Figure 3). It should also be emphasized that in clinical studies, measures should be taken to avoid the transduction of the blood antigen enzyme in normal cells, particularly red blood cells. Preferably, the gene therapy with the blood antigen enzyme could be delivered locally into cancers. Alternatively, the therapeutic enzyme is driven by a tumor-specific promoter, allowing the specific expression of the therapeutic enzyme in tumors, while sparing normal cells. This is the key issue to be considered for the safety of clinical application.

In summary, we have for the first time demonstrated the concept that the inherent blood group immune response can be explored as a potential antitumor gene therapy. Immune-mediated transfusion reactions occur if incompatible blood products are transfused into the circulation. By replacing a tumor suicide gene in a conventional gene therapy approach with a blood group antigen converting enzyme, we are able to show that breast cancer cells that express an “incompatible” blood A antigen can be readily killed by group B plasma. Transfection of blood A antigen can reduce proliferation of tumor cells and induce S phase arrest that make tumor cells more sensitive to S-phase specific chemotherapeutic agents, like 5-FU. More importantly, we also demonstrate that the appearance of blood group antigen can trigger immunogenic cell death in MDA231 tumor cells. It is assumed that the patient's pre-existing antibodies will bind to the “incompatible” antigen on the surface of tumor cells. The inherent blood group immune system in patients will destroy tumor cells through the mechanisms of cell lysis, apoptosis, and immunogenic cell death. Our study thus warrants further translational studies to apply this approach to tumor immuno-gene therapy.

## MATERIALS AND METHODS

### Cell lines and materials

Human breast cancer cell line MDA231 used in this study was purchased from American Type Culture Collection (ATCC, VA) and was maintained in RPMI 1640, supplemented with 10% fetal bovine serum (FBS), penicillin (100 U/ml), and streptomycin (100 μg/ml). Exponentially growing cells were collected by trypsin-EDTA foranalyses.

Anti blood A antigen-PE was purchased from American Research Products, (#08-9434-4, MA). Anti blood H antigen (87-N) was purchased from Santa Cruz Biotechnology (sc-52369FITC, CA). APC-conjugated Annexin V and 7-AAD antibodies were purchased from BD Biosciences (San Jose, CA). Cell proliferation reagent (WST-1) was derived from Roche (#14606400, Mannheim, Germany). Fresh plasma was obtained from breast cancer patients and healthy people who had type B blood. The study protocol was approved by the Research Ethics Board of the First Hospital of Jilin University. Informed consent was obtained from breast cancer patients. The antibodies used in this study for western blot included anti-mouse β-Actin (1:2000, Abcam, UK), Cyclin D, Cyclin E, Cyclin B1 (1:1000,abcam,UK), CDK1, CDK2, E2F, Bcl-2, P53, PARP (1:1000,Santa Cruz, USA), CDK6 (1:500,Santa Cruz, USA), ATM (1:1000,EPIT MICS,UK), p-Rb (1:1000, cell signaling, USA), BAX (1:2000, Proteintech, USA), and P21 (1:200, Santa Cruz,USA).

### Construction of lentivirus expression vector

The hABO-cDNA encoding blood group A transferase was synthesized by GenScript (Nanjing, China), based on the sequence of GenBank access number BC111575. Overlapping PCR was used to join the hABO-cDNA with the FLAG tag using the following primers:

FLAG-F15′-TAGAAGATTCTAGAGCCGCCACCATGGATTACAAGGACCACGACGGC-3′, FLAG-R1 5′-CTCGGCCATCCTGCCCTTGTCATCGTCGTCTTT G-3′, hABO-F1 5′-CGATGACAAGGGCAGGATGGCCG AGGTGTTGCGGACGCTG-3′, and hABO-R1 5′-ATT CGTCGACGATATCTTATCACGGGTTCCGGACCGCC TGGTGG-3′. The amplified hABO-FLAG fragment was digested with EcoRV and XbaI, and ligated with T4 ligase into a lentiviral vector modified based on pGreenPuro vector (SBI, CA). The pFLAG-hABO-A vector was verified by DNA sequencing.

### Lentiviral transfection of MDA231 cells

For lentivirus packaging, DNA plasmids were co-transfected with pSPAX2 and pMD2G packing vectors as previously described [38, 39]. The viral supernatants were collected at 48 hrs and 72 hrs and were concentrated for transfection in breast cancer cell line MDA231. To obtain the relatively pure clones with stable expression of blood A antigen, GFP positive clones were picked, replated in six-well plates respectively and selected by puromycin. After selection, more than 95% of cells were GFP-positive as measured by fluorescence microscope and FACS (Supplementary Figure 1). The blood A antigen expressing MDA231 cells (231-A6) were used for cell studies. MDA231 cells that carried the empty vector were used as the negative control in the study (231-C5).

### Flow cytometry analysis

For cell cycle analysis, cells were fixed by pre-cooled 70% ethanol at 4°C overnight. Before staining, the cells were washed by PBS. After incubation with 50 μl/ml PI, 100 μg/mL RNase A, and 0.2% Triton X-100 away from light for 30 minutes, cell suspension was analyzed by FACSCalibur flow cytomter (BD, CA). The cultured cells were harvested and washed twice by pre-cooled PBS. The anti-blood group antigen A and H antibodies were added to the suspended cells, that were used to quantitate the expression of blood H and A antigens. For death rate quantitation, 7-AAD was added to the suspended cells with a final concentration of 5 μg/ml. 7-AAD positive cells were defined as being dead.

### Complement dependent cytotoxicity (CDC) assay

About 2 × 10^5^ cells were resuspended in 500 μl culture medium in the presence of fresh human group B plasma (containing anti-blood type A antibody). As the control, human group B serum was inactivated by heating at 56°C for 30 min. Cells were incubated at 37°C with different concentrations of the B plasma. After washing cells with 1% BSA/PBS and binding buffer twice, APC-conjugated annexin V was added to cells with a final concentration of 5 μg/ml. The cells were incubated at room temperature in the dark for 10 minutes, washed with 1% BSA/PBS, and then 7-AAD was added to the cells with a final concentration of 5 μg/ml. Cells were analyzed using FACSCalibur flow cytomter (BD, CA) for 1 hour. We applied the parameter FSC and SSC in flow cytomety to discriminate the live cells and calculated the percentage to estimate the fraction of the complement-mediated cell death. In addition, we used Annexin V and 7-AAD together to evaluate the apoptosis of the cells as follows: apoptosis (%) = Annexin V+7-AAD-cells/all cells gated × 100%.

### WST-1 assay

Cells were resuspended at a concentration of 2 × 10^6^ cells/ml in RPMI-1640 supplemented with 10% FBS and 180 μl cells/well was seeded to a 96-well plate. After incubation for 48 h at 37°C and 5% CO_2_ atmosphere, 20 μl WST-1 solution (Cell Proliferation Reagent WST-1, Roche diagnostics, Indianapolis, USA) was added to each well, and the cells were incubated for an additional 3 hours. The plate was shaken for 20 seconds to ensure a homogeneous distribution of color. Then, the absorbance was measured using a Microplate System (BIOTEK, USA) at 440 nm. There were five wells for each group. The cell proliferation was calculated by using the following formula: Proliferation Index = (OD of the experimental group – OD of the control group/OD of the control group) x 100.

### Transwell assay

Briefly, 200 μl of cells (5 × 10^3^ cells/well) were incubated with B plasma for 4 hours and were seeded in the upper chamber of a Transwell (Corning Costar Corp., Cambridge, MA, USA), and the lower chamber was filled with complete medium before allowing the cells to migrate for 24 hrs at 37°C. Migratory cells were fixed with paraformaldehyde for 30 minutes and stained with 0.1% crystal violet solution for 20 minutes. Five fields were counted using a 20× objective (Olympus, Japan).

### Western blot

Cells were lysed using Radio Immunoprecipitation Assay (RIPA) buffer (Thermo Scientific, Rockford, IL, USA) then incubated on ice for 30 min. Then cell debris was spun down at a speed of 12,000 g for 10 min at 4°C. Concentrations of proteins in the supernatant were determined using the bicinchoninic acid (BCA) method with the protein assay kit (Beyotime, Shanghai, China). 20 μg of protein was then separated on an 8% sodium dodecyl sulfate polyacrylamide (SDS/PAGE) gel. The proteins were then transferred to a polyvinylidene fluoride membrane (PVDF, Millipore, Billerica, MA), which was blocked for 1 hr with 5% skim milk at room temperature and incubated with primary antibodies at 4°C overnight. The next day, the PVDF membrane was incubated with horseradish peroxidase (HRP) secondary antibody for 1 h at room temperature, and protein expression was then detected using the enhanced chemiluminescence (ECL) substrate kit (Amersham Biosciences, Inc.) and the all-in-one chemiluminescence imaging system (CLINIX, China).

### Tumor sphere forming assay

Cells were collected and suspended in serum-free DMEM/F12 supplemented with 20 ng/ml bFGF, 20 ng/mL EGF and 20 μl/ml B27 supplement [40]. The cells were subsequently cultured in ultra-low attachment 6-well plates (Corning Inc., NY) at a density of less than 5000 cells/well for 14 days. Spheres were observed under a microscope and images were photographed under a phase contrast fluorescence microscope (Nikon, ECLIPSE 80i).

### Assessment of the immunogenic cell death pathway

The release of ATP from vesicles were quantitated using a commercially available CellTiter-Glo Luminescent assay for ATP (Promega, Madison, Wisconsin, USA), following the protocol provided by the manufacturer.

The release of HMGB1 into cell supernatants was quantitated by an enzyme-linked immunosorbent assay (HMGB1 ELISA kit II, Shino Test Corporation, Tokyo, Japan), following the manufacturer's instructions.

### Statistical analysis

All assays were performed in triplicate. Data were analyzed using SPSS software (version 20.0; SPSS, IL). One-way *ANOVA (Bonferroni test)* was used *to* compare statistical differences for variables among treatment groups. The data were expressed as mean ± SD and results were considered statistically significant at *P* ≤ 0.05.

## SUPPLEMENTARY MATERIALS FIGURES AND TABLES



## References

[1] Bakhtiar A, Sayyad M, Rosli R, Maruyama A, Chowdhury EH (2014). Intracellular delivery of potential therapeutic genes: prospects in cancer gene therapy. Curr Gene Ther.

[2] Jakeman PG, Hills TE, Tedcastle AB, KD Di Y Fisher, Seymour LW (2015). Improved in vitro human tumor models for cancer gene therapy. Hum Gene Ther.

[3] Bhatia S, Menezes ME, Das SK, Emdad L, Dasgupta S, Wang XY, Sarkar D, Fisher PB (2013). Innovative approaches for enhancing cancer gene therapy. Discov Med.

[4] Li C, Li L, Keates AC (2012). Targeting cancer gene therapy with magnetic nanoparticles. Oncotarget.

[5] Glinka EM (2012). Eukaryotic expression vectors bearing genes encoding cytotoxic proteins for cancer gene therapy. Plasmid.

[6] Duarte S, Carle G, Faneca H, de Lima MC, Pierrefite-Carle V (2012). Suicide gene therapy in cancer: where do we stand now?. Cancer Lett.

[7] Amer MH (2014). Gene therapy for cancer: present status and future perspective. Mol Cell Ther.

[8] Rama AR, Aguilera A, Melguizo C, Caba O, Prados J (2015). Tissue Specific Promoters in Colorectal Cancer. Dis Markers.

[9] Doloff JC, Waxman DJ (2014). Adenoviral vectors for prodrug activation-based gene therapy for cancer. Anticancer Agents Med Chem.

[10] Yi BR, Choi KJ, Kim SU, Choi KC (2012). Therapeutic potential of stem cells expressing suicide genes that selectively target human breast cancer cells: evidence that they exert tumoricidal effects via tumor tropism (review). Int J Oncol.

[11] Maatta AM, Samaranayake H, Pikkarainen J, Wirth T, Yla-Herttuala S (2009). Adenovirus mediated herpes simplex virus-thymidine kinase/ganciclovir gene therapy for resectable malignant glioma. Curr Gene Ther.

[12] Ardiani A, Johnson AJ, Ruan H, Sanchez-Bonilla M, Serve K, Black ME (2012). Enzymes to die for: exploiting nucleotide metabolizing enzymes for cancer gene therapy. Curr Gene Ther.

[13] Bonini C, Mondino A (2015). Adoptive T-cell therapy for cancer: The era of engineered T cells. Eur J Immunol.

[14] Sharpe M, Mount N (2015). Genetically modified T cells in cancer therapy: opportunities and challenges. Dis Model Mech.

[15] Fujiwara H (2014). Adoptive T-cell therapy for hematological malignancies using T cells gene-modified to express tumor antigen-specific receptors. Int J Hematol.

[16] Wang L, Jin N, Schmitt A, Greiner J, Malcherek G, Hundemer M, Mani J, Hose D, Raab MS, Ho AD, Chen BA, Goldschmidt H, Schmitt M (2015). T cell-based targeted immunotherapies for patients with multiple myeloma. Int J Cancer.

[17] Figueroa JA, Reidy A, Mirandola L, Trotter K, Suvorava N, Figueroa A, Konala V, Aulakh A, Littlefield L, Grizzi F, Rahman RL, Jenkins MR, Musgrove B (2015). Chimeric antigen receptor engineering: a right step in the evolution of adoptive cellular immunotherapy. Int Rev Immunol.

[18] Tasian SK, Gardner RA (2015). CD19-redirected chimeric antigen receptor-modified T cells: a promising immunotherapy for children and adults with B-cell acute lymphoblastic leukemia (ALL). Ther Adv Hematol.

[19] Duong CP, Yong CS, Kershaw MH, Slaney CY, Darcy PK (2015). Cancer immunotherapy utilizing gene-modified T cells: From the bench to the clinic. Mol Immunol.

[20] Grupp SA, Kalos M, Barrett D, Aplenc R, Porter DL, Rheingold SR, Teachey DT, Chew A, Hauck B, Wright JF, Milone MC, Levine BL, June CH (2013). Chimeric antigen receptor-modified T cells for acute lymphoid leukemia. N Engl J Med. McLab Blanket PO# is HOA065155.

[21] Kochenderfer JN, Dudley ME, Carpenter RO, Kassim SH, Rose JJ, Telford WG, Hakim FT, Halverson DC, Fowler DH, Hardy NM, Mato AR, Hickstein DD, Gea-Banacloche JC (2013). Donor-derived CD19-targeted T cells cause regression of malignancy persisting after allogeneic hematopoietic stem cell transplantation. Blood.

[22] Elgueta R, de Vries VC, Noelle RJ (2010). The immortality of humoral immunity. Immunol Rev.

[23] Arend P (2013). Ancestral gene and “complementary” antibody dominate early ontogeny. Immunobiology.

[24] Musat M, Morris DG, Korbonits M, Grossman AB (2010). Cyclins and their related proteins in pituitary tumourigenesis. Mol Cell Endocrinol.

[25] Lim S, Kaldis P (2013). Cdks, cyclins and CKIs: roles beyond cell cycle regulation. Development.

[26] Wikman H, Kettunen E (2006). Regulation of the G1/S phase of the cell cycle and alterations in the RB pathway in human lung cancer. Expert Rev Anticancer Ther.

[27] Nigam N, Prasad S, George J, Shukla Y (2009). Lupeol induces p53 and cyclin-B-mediated G2/M arrest and targets apoptosis through activation of caspase in mouse skin. Biochem Biophys Res Commun.

[28] Kreis NN, Sanhaji M, Kramer A, Sommer K, Rodel F, Strebhardt K, Yuan J (2010). Restoration of the tumor suppressor p53 by downregulating cyclin B1 in human papillomavirus 16/18-infected cancer cells. Oncogene.

[29] Kepp O, Senovilla L, Vitale I, Vacchelli E, Adjemian S, Agostinis P, Apetoh L, Aranda F, Barnaba V, Bloy N, Bracci L, Breckpot K, Brough D (2014). Consensus guidelines for the detection of immunogenic cell death. Oncoimmunology.

[30] Kroemer G, Galluzzi L, Kepp O, Zitvogel L (2013). Immunogenic cell death in cancer therapy. Annu Rev Immunol.

[31] Krysko DV, Garg AD, Kaczmarek A, Krysko O, Agostinis P, Vandenabeele P (2012). Immunogenic cell death and DAMPs in cancer therapy. Nat Rev Cancer.

[32] Garg AD, Agostinis P (2014). ER stress, autophagy and immunogenic cell death in photodynamic therapy-induced anti-cancer immune responses. Photochem Photobiol Sci.

[33] Donnelly OG, Errington-Mais F, Steele L, Hadac E, Jennings V, Scott K, Peach H, Phillips RM, Bond J, Pandha H, Harrington K, Vile R, Russell S (2013). Measles virus causes immunogenic cell death in human melanoma. Gene Ther.

[34] Aurelian L (2016). Oncolytic viruses as immunotherapy: progress and remaining challenges. Onco Targets Ther.

[35] Bartlett DL, Liu Z, Sathaiah M, Ravindranathan R, Guo Z, He Y, Guo ZS (2013). Oncolytic viruses as therapeutic cancer vaccines. Mol Cancer.

[36] Koks CA, Garg AD, Ehrhardt M, Riva M, Vandenberk L, Boon L, De Vleeschouwer S, Agostinis P, Graf N, Van Gool SW (2015). Newcastle disease virotherapy induces long-term survival and tumor-specific immune memory in orthotopic glioma through the induction of immunogenic cell death. Int J Cancer.

[37] Gameiro SR, Jammeh ML, Wattenberg MM, Tsang KY, Ferrone S, Hodge JW (2014). Radiation-induced immunogenic modulation of tumor enhances antigen processing and calreticulin exposure, resulting in enhanced T-cell killing. Oncotarget.

[38] Zhang H, Zeitz MJ, Wang H, Niu B, Ge S, Li W, Cui J, Wang G, Qian G, Higgins MJ, Fan X, Hoffman AR, Hu JF (2014). Long noncoding RNA-mediated intrachromosomal interactions promote imprinting at the Kcnq1 locus. J Cell Biol.

[39] Wang H, Li W, Guo R, Sun J, Cui J, Wang G, Hoffman AR, Hu JF (2014). An intragenic long noncoding RNA interacts epigenetically with the RUNX1 promoter and enhancer chromatin DNA in hematopoietic malignancies. Int J Cancer.

[40] Zhao X, Liu X, Wang G, Wen X, Zhang X, Hoffman AR, Li W, Hu JF, Cui J (2016). Loss of insulin-like growth factor II imprinting is a hallmark associated with enhanced chemo/radiotherapy resistance in cancer stem cells. Oncotarget.

[41] Garg AD, Dudek-Peric AM, Romano E, Agostinis P (2015). Immunogenic cell death. Int J Dev Biol.

[42] Martins I, Wang Y, Michaud M, Ma Y, Sukkurwala AQ, Shen S, Kepp O, Metivier D, Galluzzi L, Perfettini JL, Zitvogel L, Kroemer G (2014). Molecular mechanisms of ATP secretion during immunogenic cell death. Cell Death Differ.

