# Supplementary material for: Targeted breast cancer therapy by harnessing the inherent blood group antigen immune system
Source: Oncotarget. 2017 Jan 19;8(9):15034–46. doi: 10.18632/oncotarget.14746 (PMC5362465; doi:10.18632/oncotarget.14746)
Supplement: Supplementary file 1 [file oncotarget-08-15034-s001.pdf]

Targeted breast cancer therapy by harnessing the inherent blood group antigen immune system

Supplementary Materials

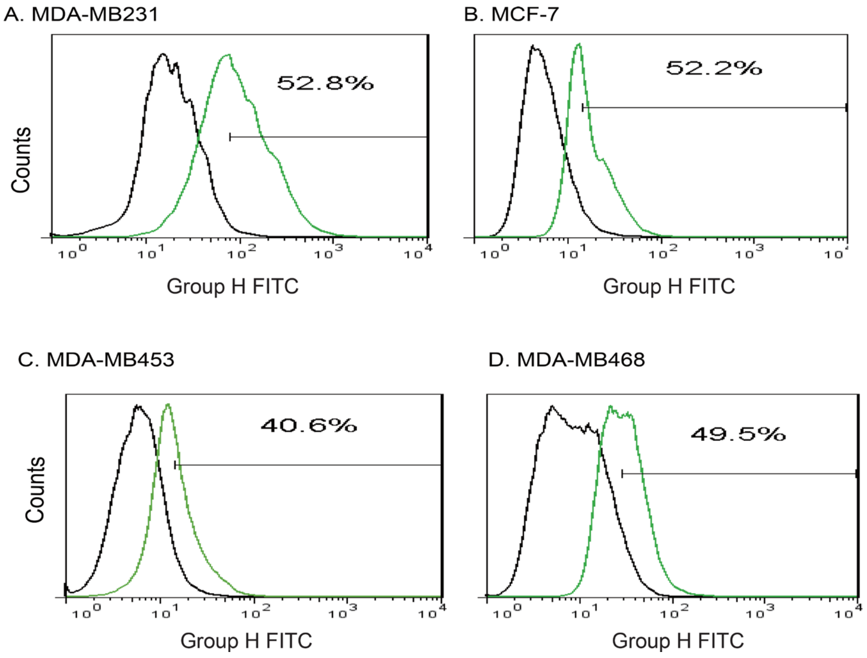

Supplementary Figure 1: Expression of the H antigen precursor in breast cancer cells. Expression of the H antigen was measured by FACS in four breast cancer cell lines: MDA-MB231 (**A**), MCF-7 (**B**), MDA-MB453 (**C**), and MDA-MB468 (**D**). The black line in the histogram represents the negative isotype control and the green line represents the fluorescence intensity of the anti-group H FITC.

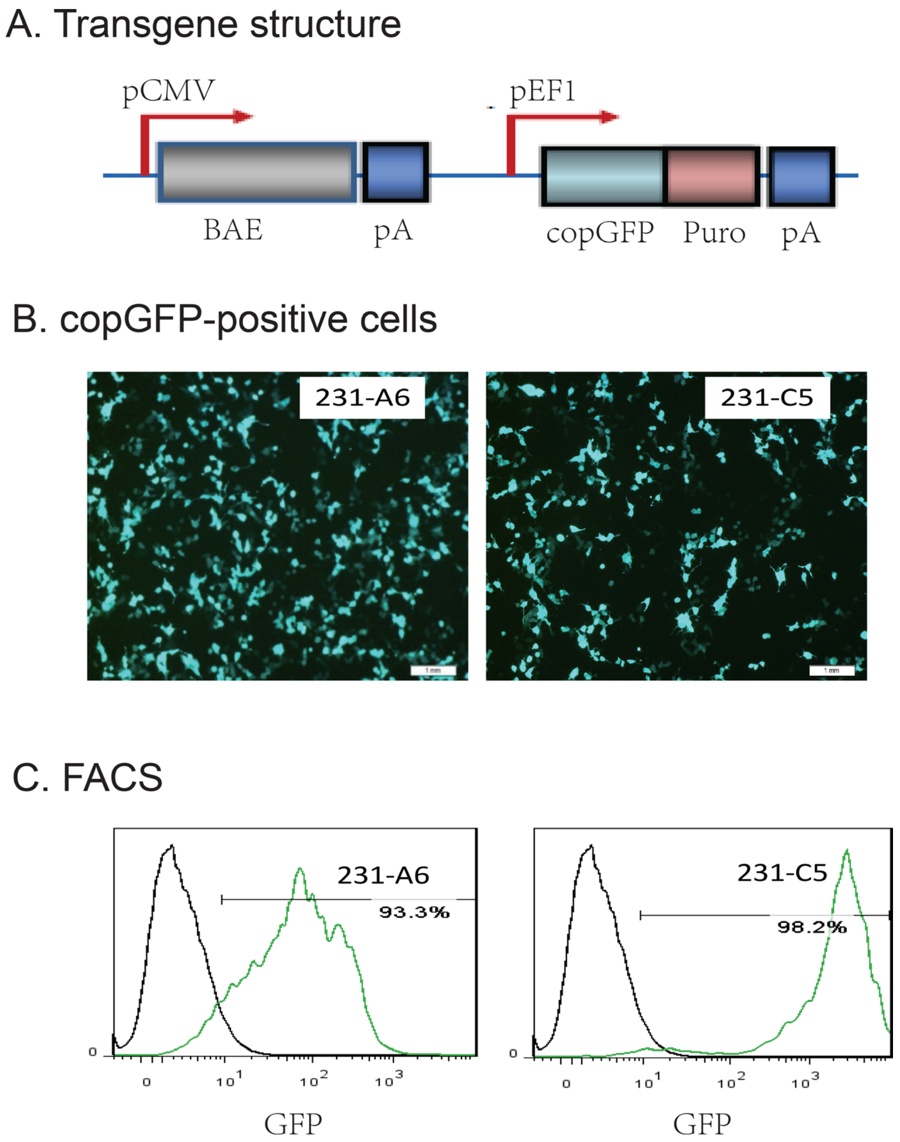

Supplementary Figure 2: Efficiency of lentiviral transduction in MDA231 tumor cells. (**A**) Transgene structure. pCMV: CMV promoter; BAE: blood antigen enzyme; copGFP: green fluorescent reporter protein; Puro: puromycin selection marker gene; pA: SV40 poly(A) signal. (**B**) Fluorescent image of copGFP-positive MDA231 tumor cells. (**C**) FACS analysis of copGFP-positive cells.

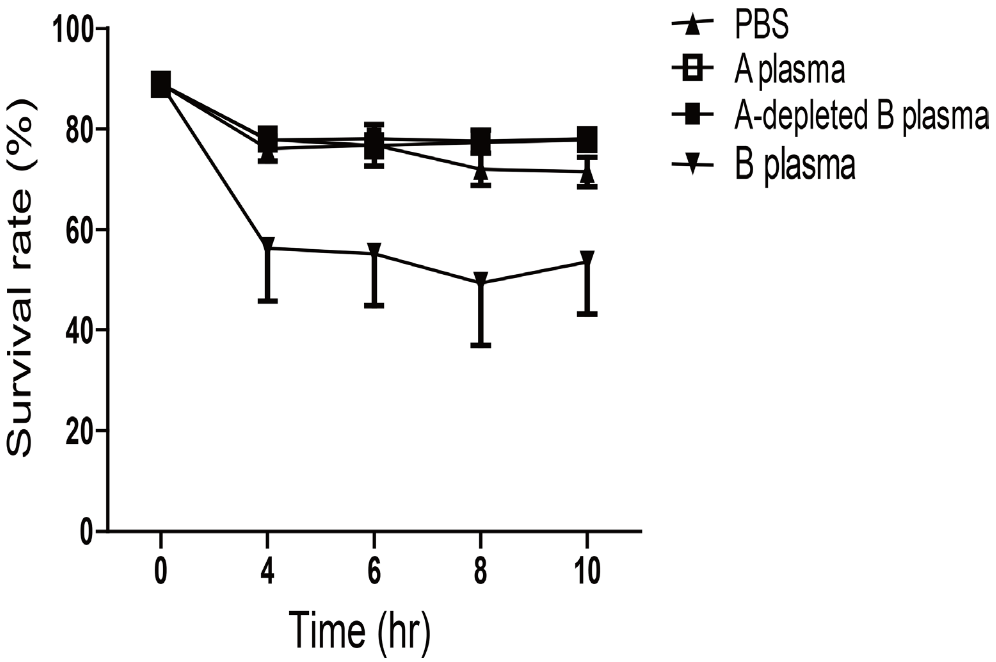

Supplementary Figure 3: Depletion of anti-A antibody in B plasma abolishes the anti-tumor activity. The role of B plasma was examined by depleting anti-A antibody in B plasma. To deplete anti-A antibodies, excess type A red blood cells (0.5 ml) was added into 2 ml fresh B plasma and incubated for 4 hours at 37°C. The A antibody-binding red blood cells were removed by centrifugation. The A antibody-depleted B plasma was then used for the survival assay. In a separate assay, type A plasma was used to replace type B plasma in the assay. A plasma: plasma derived from a healthy subject with A blood type; A-depleted B plasma: B plasma in which anti-A antibody has been removed; PBS: negative control; Note the loss of anti-tumor effect in both A plasma and the anti-A antibody-depleted B plasma.

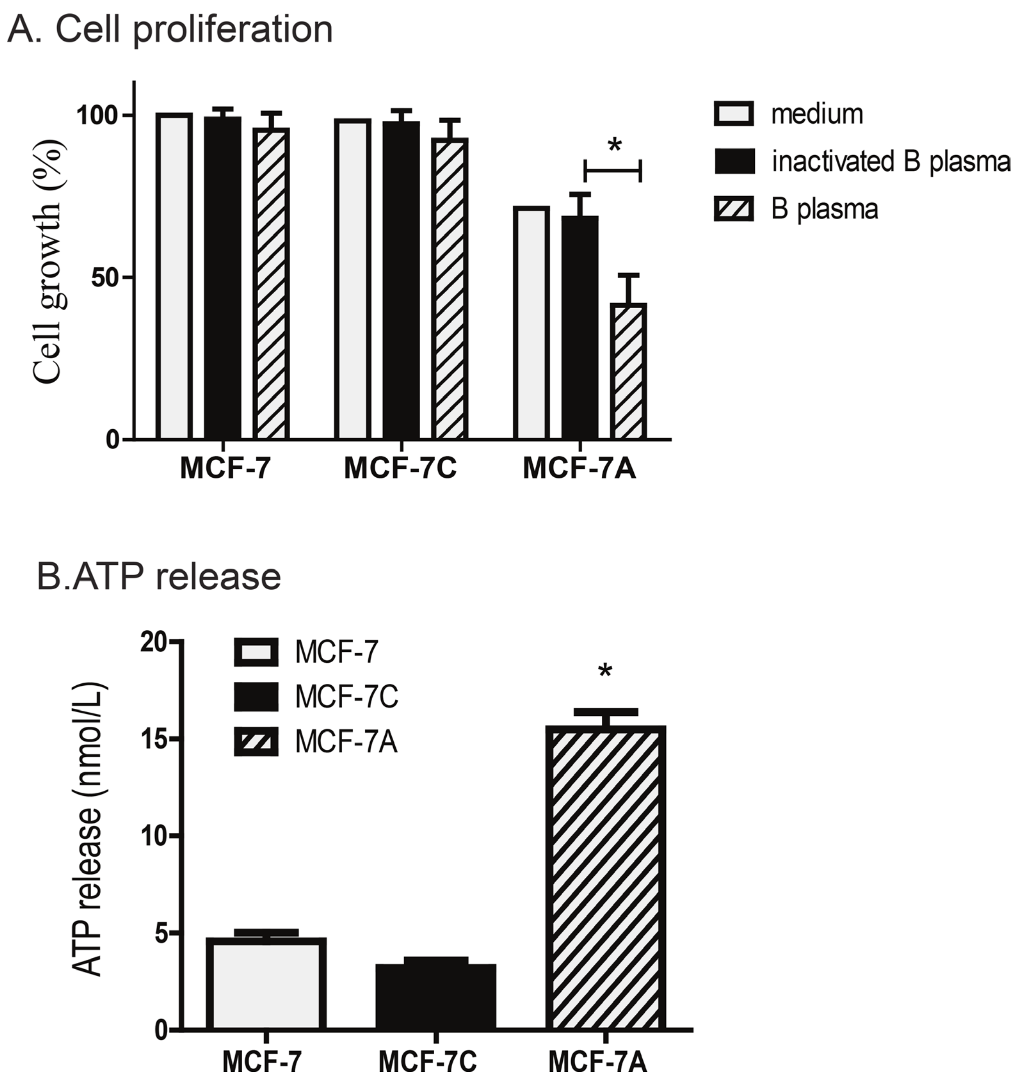

Supplementary Figure 4: Blood type antigen therapy in MCF-7 breast cancer cells. (**A**) Cell proliferation as measured by WST-1 assay. Cells were treated with 5% B plasma for four hours. Forty-eight hours following plasma treatment, cells were collected for measurement of cell growth. Inactivated group B plasma was used as the assay control. *$p < 0.05$ between the inactivated B plasma and the B plasma groups. (**B**) Release of cellular ATP in the medium. Immunogenic cell death was quantitated in MCF-7 breast cancer cells that stably express the group A antigen. The data are the mean±standard deviation of three independent experiments. *$p < 0.05$ compared with the control groups.
